# Disentangling the clinical data chaos: User-centered interface system design for trauma centers

**DOI:** 10.1371/journal.pone.0251140

**Published:** 2021-05-12

**Authors:** JaeYeon Park, Soyoung Rhim, Kyungsik Han, JeongGil Ko

**Affiliations:** 1 School of Integrated Technology, Yonsei University, Incheon, South Korea; 2 Department of Computer Engineering, Ajou University, Suwon, South Korea; University of Colorado-Denver, Anschultz Medical Campus, UNITED STATES

## Abstract

This paper presents a year-long study of our project, aiming at (1) understanding the work practices of clinical staff in trauma intensive care units (TICUs) at a trauma center, with respect to their usage of clinical data interface systems, and (2) developing and evaluating an intuitive and user-centered clinical data interface system for their TICU environments. Based on a long-term field study in an urban trauma center that involved observation-, interview-, and survey-based studies to understand our target users and their working environment, we designed and implemented *MediSenseView* as a working prototype. *MediSenseView* is a clinical-data interface system, which was developed through the identification of three core challenges of existing interface system use in a trauma care unit—*device separation*, *usage inefficiency*, and *system immobility*—from the perspectives of three staff groups in our target environment (i.e., doctors, clinical nurses and research nurses), and through an iterative design study. The results from our pilot deployment of *MediSenseView* and a user study performed with 28 trauma center staff members highlight their work efficiency and satisfaction with *MediSenseView* compared to existing clinical data interface systems in the hospital.

## 1 Introduction

From patients’ physiological signal data to lab test results, medical images and electronic medical records (EMRs) (All abbreviations used in this paper are explained in Table 5), today’s hospitals are overwhelmed with patient data [1, 2]. Such data, generated at massive quantities in most major hospitals, is an important key that determines the quality of patient care experience, given that many clinical decisions are based on the careful analysis of patient data collected from various medical devices, and on the results obtained from different computer software that supports such clinical data analysis [3–6].

**Fig 1:**
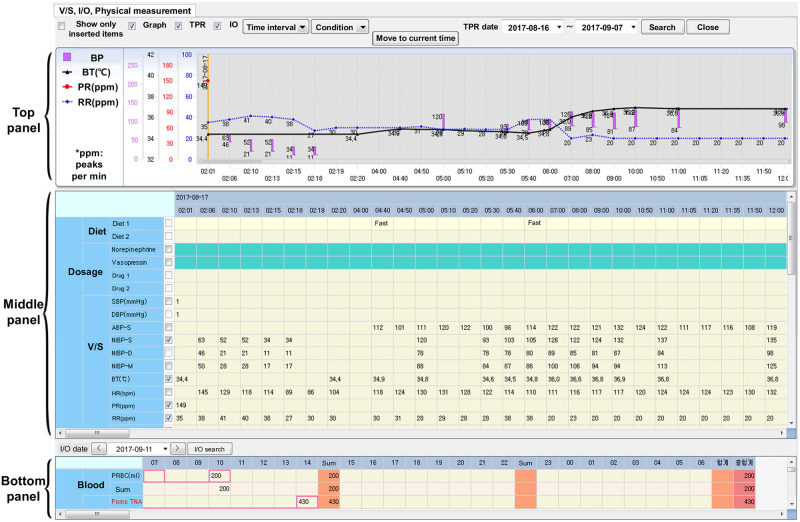
The Electronic Medical Record (EMR) interface system currently being used in our target hospital’s trauma center. This interface consists of three panels: top, middle, and bottom. The top panel plots graphs of a patient’s numerical clinical data shown in the middle panel. The bottom panel displays a patient’s dietary intake information (e.g., meals and drugs).

While some departments within a hospital can enjoy the luxury of reviewing patient charts over longer periods of time, emergency care units, such as a trauma center, follow a set of very time-critical protocols. In trauma intensive care units (TICUs), it is of utmost importance that the clinical staff as summarized in Table 1 can *quickly* and *comprehensively* access the patients’ data. Effective user interfaces that “well-expose” clinical data can allow medical personnel to make swift, and accurate decisions. For this, patients at the TICU are very closely monitored and the data generated from them are typically gathered in an EMR. The EMR provides an interface for the staff to observe and access the collected patient information. Fig 1 presents a sample snapshot of the EMR user interface currently being used in our target clinical environment.

**Table 1:** Summary of user groups’ seniority, specialty, and role at an urban university hospital’s trauma center. We introduce how each user’s role is different following their daily routine in Section 4.

	Number of participants	Position	Specialty	Role
Clinical doctor	7	Associate professor (1) Assistant professor (6)	Trauma surgery (abdominal trauma and thoracic trauma)	Patient rounds to check patient status and manage emergency events based on 24-hour shifts. Mortality report meetings and ICU patients to identify the effectiveness of their decisions.
Clinical nurse	10	Chief nurse (3) Others (7)	Clinical nurse practitioner Emergency nursing	Take care of patients in the ICU including changing the clothing, posture correction, and verbal verification of patient status. Prepare patient for clinical procedures (e.g., operations, CT or MRI images taking, etc.). Record all activity results and patient status to EMR. (e.g., vital signs, body temperature, medical sensor data).
Research nurse	17	Research assistant (17)	Clinical nurse practitioner Emergency nursing	Support clinical research tasks (e.g., clinical data analysis, IRB, clinical survey, etc.). Research activity with doctors based mainly on EMR information and physiological signals.

TICUs are typically crowded with hundreds of severe patients every week with only a limited number of clinical staff. Clinical protocols here are considered to be intense, and decisions on operations or patient diagnosis need to be made with minimal hesitation to increase the chances of saving patients’ lives. Under such circumstances, the clinical staff are also suffering from high levels of manual administrative work [7]. This can dwindle their patient care times and threaten patient safety in the worst case [8]. To make things worse, many of the clinical procedures and computer interfaces in TICUs are surprisingly inefficient [9–11].

To confirm such inefficiency in data access and the workflow, we started this work by performing a field study which includes interviews, surveys and observational studies, with clinical and research staff at an urban university hospital’s trauma center. This trauma center, the largest in the province and second largest in the nation, treats 2,700 patients on a yearly basis. Through a half-year-long field study with a fully operating prototype connected to the real-time data available through the hospital’s database, we identified how clinical and research staff at the trauma center access and analyze patient data using the existing EMR system developed by Metanet DT [12], a local mid-sized software development firm, which connects to the Philips IntelliVue MX800 [13] patient monitoring device.

From this study, we identified core design requirements and interface functionalities that can help improve the clinical procedures for trauma care. Specifically, we identified that three core tasks need to be solved to address the problems with the current-day clinical data interfaces: **(1)** *data integration* from heterogeneous devices, **(2)** *automated administration* activities, and **(3)** *mobile interface* system support. In detail, we recognized that the EMR was not the only source of decision-support data in the trauma center, but the patient monitoring devices for collecting physiological signals also play an essential role. Data streams of these two heterogeneous systems are only lightly-coupled, but both systems are actively used to gain comprehensive knowledge of a patient. The integration of these data sources can help improve efficiency by allowing prompt decision making. Furthermore, there are a pile of manual administrative work to handle, such as printing abnormal physiological signals on paper and digitizing them as records for future use. Such routine processes can also be improved by an efficient system that well-integrates data and presents them in a form that supports the work protocols. Finally, since doctors are often away from a patient to serve other patients or attend meetings, in cases of emergency, allowing them to observe patient data using mobile devices can assist in making more accurate and quick clinical decisions. Overall, while trauma ICU protocols have dramatically improved over the years with the help of state-of-the-art monitoring devices, we believe that these devices were designed without enough consideration on the clinical protocols and real-world usability; especially for such a special environment as a trauma care unit.

Based on such observations, we designed *MediSenseView*, a user-centered system for interfacing clinical data. *MediSenseView* integrates data from both the EMR and physiological signal monitors (patient monitors), and simplifies many of the routine manual tasks that the clinical staff face, while offering effective mobile interfaces. Using iterative design studies, we improved the design and functionalities of *MediSenseView* through three design meetings with six clinical/research staff in a trauma center. Together with a group of 28 trauma center staff members, we conduct pre- and post-surveys to examine whether *MediSenseView* can make their clinical work easier and more efficient compared to the existing devices (e.g., EMR and patient monitor systems). Our study results show the clinical staff’s overall positive responses to *MediSenseView* and confirm that it meets their expectations as an effective interface system for trauma center environments.

Specifically, the main contributions of our work can be summarized in three-fold.

- **[Understanding the users and their work practices]** We performed a series of interviews and surveys, along with a half-year-long observational study with members of an urban trauma center to identify the usage patterns of clinical data in a TICU, and compiled a comprehensive set of user requirements for designing an effective user interface for trauma care units.
- **[User-centered system design]** We designed *MediSenseView*, an interface system that integrates real-time data collected from patient monitors and the hospital’s EMR database. The system was carefully designed based on the requirements and functional requests made by TICU staff, and was revised with respect to their feedback through iterative meetings.
- **[System evaluation]** We evaluated *MediSenseView* through a pilot deployment that involves 28 trauma center staff members. Results and feedback from this study suggest that the satisfaction levels of TICU staff are high and the users are satisfied with the user-centered interface that *MediSenseView* provides.

The remainder of this paper is structured as follows. In the next section, we first discuss related work and position our work in the existing literature. Next, we introduce our observational and pre-survey studies for understanding the target users and their working environments in Sections 3 and 4. These studies lead us to define a set of problem statements in Section 5, and to address these problems, in Section 6 we present *MediSenseView* by introducing its iterative design process. We then evaluate the performance of *MediSenseView* through another series of user studies in Section 7. Finally, we bring up interesting discussion topics by leveraging our year-long study experience in Section 8 and Section 9 concludes this work.

## 2 Related work

### 2.1 Computer systems without user-centered design

While recent human-computer interaction (HCI) research has shown that computer supported cooperative work (CSCW) studies can be effective in healthcare applications, many studies are still conducted without a deep understanding of the users, their interactive relationships and system-, usage-requirements. Representative studies such as LifeLines [14] and LifeLines2 [15] were designed to provide a general visualization environment for personal medical history. AnamneVis [16] formats results extracted from medical diagnostics in a hospital into the Five W model and passes it on to the visualization engine which has all procedures and data models to encode the ‘Five Ws’ (who, what, where, when, and why) into the corresponding visuals and interaction procedures. KHOSPAD [17, 18] presents medical events with support for controlling temporal granularity and indeterminacy to improve the quality of the care protocol process. KHOSPAD provides an environment to represent complex notations for events and offer additional viewpoints on temporal relationships between events. PatternFinder [19] adds the ability to search multiple patient records. The novelty of PatternFinder lies on its query specification for temporally ordered events with value and time span constraints. PHiP [20] is designed as a clinical interface suitable for mobile devices (e.g., PDA). For epilepsy patients, PHiP provides a display showing the patients’ history and the staff can visually query patient data within the hospital data. PHiP was evaluated through interviews with neurologists to confirm that the designed interface was a valid support to neurologists’ activities. Some systems provide categorized abstractions for raw numeric data. VISITORS [21] visualizes numerical data using a combination of point plots and line charts. The visual presentations of VISITORS allows the overlay all patients’ data points on the same coordinate space. For patient monitoring, WBIVS [22] offers a web-based interactive visualization system to show both numerical and categorical pulmonary data. WBIVS provides combined line plots for multiple numerical variables and matrix plots for categorical variables for observation and patient monitoring. While these studies have been shown to be effective tools for clinical environments, the design of these systems were not influenced by the inputs provided by the actual users of the systems. Savoy et al. [23] designed a graphical user interface (GUI)-based cognitive system for primary care providers to improve the currently used GUI’s inefficiencies in information exchange, care-coordination, and inter-physician referral. Specifically, their GUI interface was designed through (i) an understanding consultation process and (ii) interviews and observations to understand design requirements. Finally, the authors tested their prototype system with 30 physicians. Day et al. [24] designed MyChart, mobile applications for reducing the complexity and time-consuming process in shared decision-making between a physician and a patient. MyChart enables secure bidirectional data exchange and data reading/writing on a standard EMR in real-time. A usability study with nine cases was reported regarding prostate-specific antigen testing related decision-making for prostate cancer screening. Cai et al. [25] introduced SMILY, a human-AI collaborative decision-making interface that provides interactive refinement search on similar images related to related diseases. Deep learning-based SMILY allows explainable and understandable disease tracking compared to existing algorithmic methods. Harris et al. [26] proposed the Critical Care Health Informatics Collaborative (CCHIC), an integrated multi-center database for electronic health records (EHR) from the ICU. The purpose of CCHIC was to build a valuable clinical database and provide data to clinical researchers through a scalable EHR processing pipeline. The Integrated System for Multimodal Data Acquisition and Analysis (INSMA) [27] was also proposed to offer physiological data generated from various medical devices. As a bedside monitoring device, INSMA provides real-time information for improved decision making via acquisition, parsing, and visualization modules.

### 2.2 Role of human-computer interaction in clinical system development

Technologically advanced clinical systems are prevalent in the ICU to help clinical staff effectively monitor patients [28]. However, there exist many barriers to clinical system innovation. According to the World Health Organization [29], while the diffusion of health care innovation has already begun, challenges still lie in utilizing or reforming clinical systems, due to the lack of consideration in many contextual factors (e.g., personal, environmental, cultural) and of inadequate guidelines for clinical systems (e.g., not enough copies of user manuals for all users, direct links broken between producer and end users, and lack of technical expertise and information for maintaining or using clinical devices). A primary reason for this issue relates to the absence of understanding users, environments, and contexts when designing systems for clinical environments [30].

User-centered design (UCD) is an iterative design process in which system designers focus on the current or potential users and their needs in each phase of the design process [31, 32]. UCD not only leads to practical guidelines and a set of evaluation criteria of a newly designed system [33], but also calls for involving users throughout the design process via a variety of research and design techniques to create highly usable and accessible products [34].

The core purpose of clinical devices is to provide clinical staff with the ability to better monitor and treat the patient [35]. Thus, when applying UCD to the medical system development domain, the first step is to explore clinical processes and workflows of clinical staff and understand how clinical systems are used. During this phase, system designers need to examine whether the current design or the use of the clinical systems sufficiently satisfies the needs of the clinical staff, regarding their work efficiency and perceived satisfaction. Once identifying issues and challenges of the use of clinical systems through clinicians’ points of view, involving the staff in the design process of medical systems becomes important [35, 36]. The end product from UCD will not only improve work efficiency but also reduce user errors, costs, time, and increase work satisfaction, which are all critical factors to consider in the clinical application domain. For these reasons, we adopted UCD for the design and development of *MediSenseView*.

### 2.3 Novel hospital systems with user-centered design

Due to the importance of maximizing the efficiency of cooperative activities in clinical work protocols and practices at large-scale hospitals, many studies in medical settings have been examined from various perspectives such as utilization of artifacts and technology, location- and time-critical cooperative work, extracting and expanding contexts from medical staff, and designing supportive systems for these personnel [37]. Specifically, to capture an in-depth understanding of the complex interactions among clinicians and their cooperative works, our study includes a ‘workplace study’ within the unique hospital environment. Such an approach is in-line with most of the previous work that emphasize the importance of understanding system usage characteristics ‘on-site’ [38, 39].

As a series of recent notable studies in general hospital wards, Bardram et al. [40–43] built a novel data-focused framework, Activity-Based Computing (ABC), which enables the integration of various tools to address cooperation in human work activities [44], based on 11 workshops with observations and interviews for theoretical and empirical understanding. In order to effectively support multitasking, mobility, and collaborative work via generic activity representation integrated from various tools, the ABC framework focuses not only on improving data access for computerized information (e.g., picture archiving and communication system (PACS), electronic medical record (EMR)) but also non-computerized artifacts that represent the heterogeneous roles and functions in a hospital ward. As for other works, there have been in-hospital studies for improving data integration and data access for supporting cooperative work by applying HCI elements. Lifelines2 [15] and Similan [45] propose interactive visualization systems to identify and examine event sequences in multiple and categorized patients’ data from the hospital’s electronic health record (EHR). Similarly, based on a list of categorized variables, they use patient records vertically stacked on a shared horizontal timeline. The authors base their work on a series of qualitative studies in the process of designing these systems.

Kyng et al. [46] focused on emergency situations and activities that occur at incident sites and derived design elements with participatory design approaches in designing interactive systems for emergency response events. Specifically related to emergency situations, the authors show conclusive findings and practical approaches to support the importance of communication and information flow for team situation awareness and effective teamwork. The AWARE architecture from Bardram and Hansen et al. [9, 47, 48] and Hansen et al. [49] moved their focus towards the operation ward to efficiently communicate with involved clinicians and support the distributed collaboration and coordination of clinical work. In detail, through long term studies including brainstorming sessions and workshops, logging and survey, and observation, AWARE-based systems for intimate communication lead to efficient cooperative work in an operating room. In an environment such as the operating room which requires close communication, improving a trauma resuscitation team’s performance should be based on understanding the causes of human errors at the trauma bay. Parush et al. [50] observed situation-related communications that were susceptible to breakdown in an operating room and determined the display requirements on the computer interface. Additionally, Parlak et al. [51] studied a way to efficiently interact with a diverse workforce at all staff levels in resuscitation teams at a trauma bay. Through task, contents, and video analysis, Parlak et al. studied domain tasks and procedures to identify activities and objects that require tracking in a trauma resuscitation room. More recently, Kusunoki et al. [52] proposed ideas for technological innovation to support ad-hoc, multidisciplinary medical teamwork during trauma resuscitation based on participatory design. Through the study process (process-based vs. status-based designs and role-based vs. team-based displays) they derived requirements for clinical participants related to the team-based interface in the trauma center. Our work shares many commonalities in how the study is conducted. Specifically, the understanding of various stakeholders and their work activities is considered when designing an effective interface for the target environment. For this, our work conducts observations, pre-surveys, and interviews to better understand each group. Based on this understanding, we carried out the system design phase to identify the needs of the potential users to reflect them on our end-system, which is a similar approach to Kusunoki et al. Nevertheless, our work is tailored towards a more general environment than what Kusunoki et al. have targeted for. We target to present interface designs for the entire TICU, while Kusunoki et al.’s work focuses on the operating room. While both environments represent urgent clinical settings, varying requirements can be gathered due to the different use of computing interfaces. Our work focuses on re-exploiting the existing interfaces so that it meets the specific needs of TICU environments. Furthermore, on a functional perspective, our system includes the use of real-time physiological signal data and deals deeply with how the integration of heterogeneous data points can impact clinical work protocols.

In summary, we notice that many clinical systems were designed for patients and caregivers to access information on the patients’ conditions. Most of these studies have developed a completely new interface based on clinical staffs’ needs derived from a UCD process. However, the integration of new systems can cause issues due to the learning curve of computer systems by non-computing professionals [53–55]. Therefore, our work identifies the needs of clinical staff with a focus on *improving* the existing system based on the UCD methods as described in Vredenburg et al. [33] (neglecting some specific methods such as card sorting and participatory design) rather than developing a new interface from scratch. Especially for providing emergency medical services, with clinical staff operating under time pressure, we have identified that existing systems can be problematic as information is transferred via handwritten notes [56] or bottlenecks in patient flow can arise from the additional time spent on activities surrounding EMR use [57]. In identifying and improving such issues with the existing systems, our work attempts to find a way to design/implement an *improved version* of the existing system that suits the workflow of clinical staff.

Relatively few systems were designed based on the understanding of core clinical user groups (i.e., doctors, nurses) and the clinical protocols of each, and even such studies mostly lack detailed user-studies that involve a concretely designed/implemented system, a proper deployment, and a phase of gathering requirements and feedback from the clinical staff. This work will present a “user-centered” clinical staff supporting system, in which the system design requirements were gathered from the users of the system, and the resulting system was iteratively evaluated by the target staff.

## 3 Overall workflow of our study

The goal of this work is to provide an improved interface system for accessing clinical data in a trauma care center’s trauma ICU (TICU).

**Fig 2:**
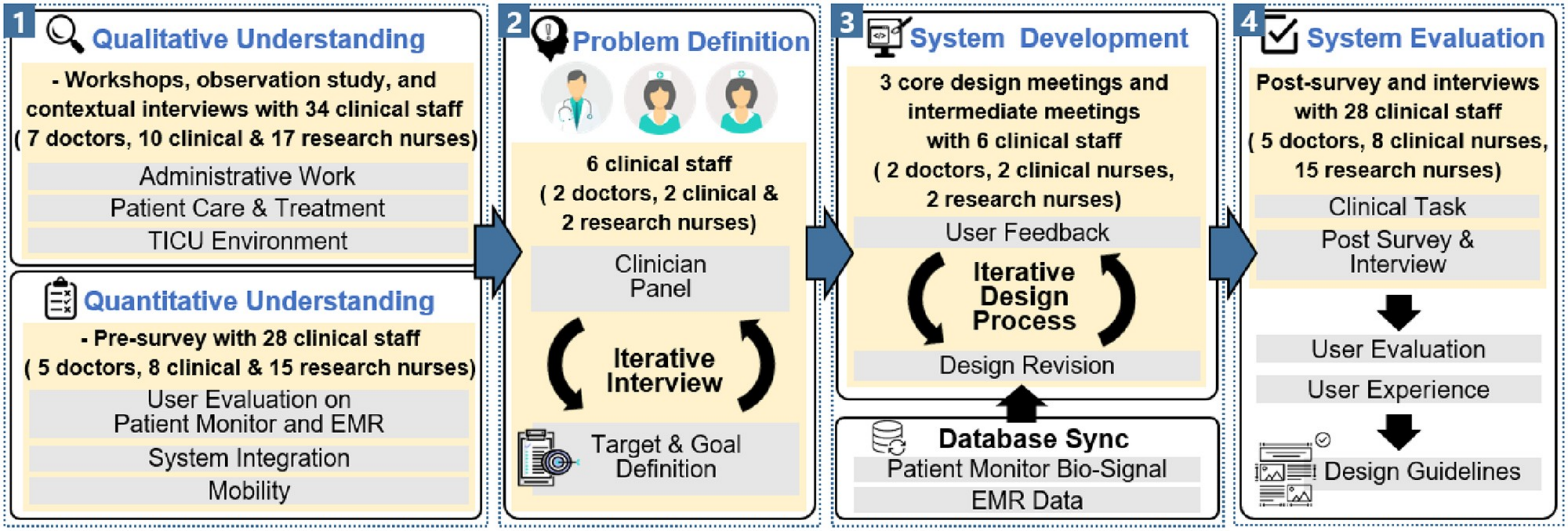
The overall workflow of our study, consisting of (1) the understanding of the TICU work environment (through qualitative and quantitative studies), (2) problem definition, (3) system development, and (4) system evaluation steps.

Fig 2 illustrates the overall workflow of how our system, *MediSenseView*, is designed. Specifically, our workflow consists of four main steps, and we highlight that *MediSenseView* is designed based on systematic steps and reflects clinical staff’s requirements and work practices. Our studies were carefully reviewed and approved by the Institutional Bioethics Committee of the internal Institutional Review Board (IRB) at the target university hospital. While we detail each of the four steps of our study in the following sections (i.e., Sections 4, 5, 6, and 7), below, we present a short overview of each study procedure depicted in Fig 2.

### 3.1 Step 1: Understanding the trauma intensive care unit work environment

This phase started with a half-year observation study, which involved 34 TICU staff members (7 doctors, 10 clinical and 17 research nurses) in an urban university hospital. Our target hospital serves 2,700 patients per year and is the largest trauma center in the province (and second largest in the nation). In this phase, we held two workshops in which a total of 34 clinical staff (7 doctors, 10 clinical and 17 research nurses) from the TICU participated. Here we focused on learning the trauma center’s clinical protocols, understanding the roles of different staff member groups, and identifying how clinical and research staff at the hospital access different types of patient-related data.

In detail, these workshops helped us to understand the interconnected clinical protocol that each group proceeds with different purposes. Such understandings of the protocol will help us to materialize a component into system design. After the workshop, the topics covered in the workshop were used as guidance for contextual interviews to understand the topics in more detail. Some examples are as follows.

“… Through the EMR interface, we only check physiological signal trends. If we need detailed information on the signals, we check the patient monitor device separately. We also record specific disease events based on the EMR interface, so we cannot record detailed ECG signals. While the doctors want detailed continuous ECG information, we can not record ECG signals from the EMR.”

*(Clinical nurse A)*

“… However, we don’t have the authority on this record. So we simply hope the clinical nurses record the patient’s data as often as possible. Sometimes, the graphs in the EMR at the top panel are not very informative or not easy to identify data for a specific event.”

*(Doctor A)*

“… In terms of conducting clinical research, we want to identify not only the trend of a patient’s data but also the detailed data including various physiological signals at the same time.”

*(Research nurse A)*

After the workshop, the discussions from the workshop were used as information that form a contextual interview with each staff group to deeply understand their issues on the current interface systems. In addition to the workshop meetings, the observation study also involved daily interaction and an average of four short meetings every week with the medical personnel to monitor how the staff used the existing interfaces. During this study, we followed the staff members closely throughout their daily work activities (e.g., morning rounds, meetings, operations, routine patient care) and hand/voice-recorded how they interacted with various clinical data interfaces and what interfaces were used for which clinical purpose to analyze our observations thoroughly. In addition, we conducted a survey and a series of contextual interviews with 28 clinical staff (among the 34 above) to understand their perceptions and experiences related to the ease-of-use, work efficiency, necessity of improvement, and system integration for the existing computer interfaces. Among the 34 participants that took place in the workshops, we note that 28 of them performed the contextual interview and post- & pre-survey. The six participants that were excluded from the interviews and surveys were all part of a *clinical panel* (two doctors, two clinical nurses, and two research nurses) that provided us with detailed suggestions on the interface design. The reason for this is that the panel members are highly experienced clinical staff regarding their workflow in each group, and in Fig 2 it is necessary to elicit objectives and non-prejudicial problem definitions in the problem definition process. We offer additional detailed information on our contextual interviews in Section 4.1.

### 3.2 Step 2: Problem definition

In this phase of our study, the issues with the existing clinical data interface systems identified in the previous phase were discussed with our trauma center panel members (who did not take part in the survey mentioned above), which consisted of two doctors, two clinical nurses and two research nurses who work at the trauma center. All panel members are extremely experienced in their respective fields and have worked in the trauma center for more than 10 years. The panel members were selected based on their work expertise and also on their authority in making urgent clinical decisions or designing research directions. They were the most suitable group of staff members to offer us with a high-level view of the issues that each user group were facing, which helped us to shape the problem definition and system design for this study. These panel members were excluded from the contextual interviews and surveys so that their participation did not bias the evaluation results. Through an iterative interview process with the panel, we were able to define a set of concrete problem statements and a rich set of user requirements that helped shape out the functionalities and design of a system prototype for clinical data interfacing. Additionally, we continuously gathered opinions from other clinical staff at the trauma center to identify the practical issues that they are facing and needed to be solved.

### 3.3 Step 3: System design and development

In this phase, we implemented the initial design of *MediSenseView* and held regular meetings with the potential users of the system. Specifically, following the process of user-centered design (UCD), we held three core design meetings with the trauma center panel members. Through these meetings, we revised the design and functionality of *MediSenseView* through an iterative design process. Specifically, during each meeting, we presented a working prototype of *MediSenseView*, explained its core features and how each feature addressed their needs. Their feedback was received and addressed in the next design phase, and we repeated this revision process three times which was the point in which everyone agreed that *MediSenseView* successfully reflected the core features and the evaluation of *MediSenseView* would be meaningful.

Additionally, in this phase, we note that we worked tightly with not only the trauma center staff members, but also with the hospital’s IT management team. This was to secure a tight and reliable connection and synchronization between *MediSenseView* and the hospital’s EMR/physiological signal database, given that such integration was essential in designing a solid working prototype system.

### 3.4 Step 4: System evaluation

Finally, in the system evaluation phase, we conducted a post-study based on a pilot deployment study of *MediSenseView* with a group of 5 doctors, 8 clinical nurses and 15 research nurses to evaluate their overall user experience and work efficiency when using *MediSenseView*. Based on the survey results, we analyzed the improvements and differences between the pre- and post-studies, and conducted interviews with the study participants to understand any challenges in using *MediSenseView* to articulate additional design implications and guidelines.

For analyzing the interviews, three authors of this paper (two computer scientists and one clinical professional) carefully coded the interview transcripts. The task was to go through the transcripts looking for where the clinical staff mentioned anything about their experience and work practices with the current clinical data interface system. These fragments were then coded by reference to particular ideas or phrases mentioned in the text. The inter-coder reliability was verified via Cohen’s Kappa measurement [58]. Specifically, Cohen’s Kappa score for each main/sub-category was 0.85, indicating that the inter-coder reliability was close to “almost perfect”.

### 3.5 Notes on our testing methodology

As we will detail later, all surveys conducted in this work, both pre- and post-surveys, were performed over the same set of trauma center clinical staff. The pre-surveys convey information on the staff-perceived limitations of the currently used interface system. The results from the pre-survey were the motivation for a new interface system and set the requirements on its functionalities.

On the other hand, the post-survey was conducted after the trauma center clinical staff experienced the final version of our proposed interface system under a fully operational scenario for 1.5 months. The goal of this post-survey was to confirm that *MediSenseView* achieves satisfactory performance in meeting the requirements and that it integrates well with the clinical protocols that each staff member performs.

Unfortunately, given that the set of survey questions are designed differently for different purposes, direct computation on statistics on the *improvement in usability* cannot be computed with existing quantitative metrics. Nevertheless, as the following sections will detail, we argue that these two sets of independent surveys are useful in its own and sufficient in demonstrating the effectiveness of *MediSenseView* as a new interface system tailored for trauma center clinical protocols.

## 4 Understanding the trauma intensive care unit work environment

Understanding the users of our system and their work environment is important given that this environment and their work tasks should be the basis of a well-designed system [59]. We realized that on a clinical data interfacing system’s perspective, the trauma care unit in a university hospital, our target environment, had three core user groups: *doctors*, *clinical nurses*, and *research nurses*. These different groups utilize and contribute to the clinical data in different ways; thus, complicating the system design. To understand their work protocols and their tasks throughout the day, we performed a combination of observational and survey studies to qualitatively and quantitatively understand their needs.

### 4.1 Understanding the three types of staff members (User groups) in the trauma care unit

#### 4.1.1 Clinical doctors

Clinical doctors in trauma centers work based on 24-hour shifts. In the morning, they perform morning rounds, in which they receive the nocturnal reports on each patient’s status and disease/emergency events occurred from night-shift clinicians and physically observe each patient to monitor their conditions. Doctors use the EMR for examining nursing records and the trend of physiological signals of a patient. They also use the patient monitor device to gather detailed perspectives on the general status or emergency events. After performing their rounds, the doctors gather for mortality report meetings to analyze the records of the patients who died (if any) to review the effectiveness of their clinical decisions. In the afternoon, a subset of the doctors have outpatient care duties at general wards (patients discharged from ICUs), and others take part in surgery or take care of ICU patients. During their daily routine, doctors receive many calls from other clinical staff, in which clinical nurses notify the doctors of making urgent clinical decisions or treatment plans. In addition to the roles above, given that the trauma center is part of a university hospital, the doctors are also under pressure to perform clinical research. For this, they analyze the patient data and identify interesting incidents that are reportable to their research community.

#### 4.1.2 Clinical nurses

In the TICU, clinical nurses work based on 8-hour shifts. Working hours are divided into the morning/afternoon, the afternoon/evening, and the night/morning times. Ten nurses, including three chief nurses, work at each shift. When a patient experiences emergency conditions or when a patient moves from the ICU for operations or image taking (e.g., CT, MRI), nurses take the role of preparing the patient for the clinical activities. In addition to the emergency events, the clinical nurses also consistently take care of the patients in the ICU. This job includes changing patients’ clothes, posture correction, and verbal verification of patient statuses. All of these activity results are recorded to the EMR, and the clinical nurses also manually measure additional sensor data (e.g., body temperature), keep track of medication history, and record this information to the EMR as well. Furthermore, clinical nurses manage the paperwork for archiving patient data. For example, the process of ECG scanning, in which the clinical nurses print out ECG records of notable events and paste this information on a sheet for later review, is a routine administrative task that they perform.

#### 4.1.3 Research nurses

While clinical nurses support the clinical tasks of the doctors, research nurses perform research with the doctors at the trauma center. Their job is mostly related to analyzing EMR data and (less frequently) physiological signal data. In the TICU, research nurses start their day by gathering patients data through the EMR for analysis. With this data, the research nurses perform data pre-processing for deeper data analysis. Statistical information is frequently reported to the doctors to discuss and specify study directions. These research nurses also take care of administrative documentation such as IRB application writing and survey document design. If data from the EMR is insufficient, the research nurses directly approach the patients at the TICU to gather or validate their results.

#### 4.1.4 Lessons from understanding the three user groups

Overall, our observational studies at the trauma center revealed that the three user groups access and utilize clinical data in different ways for different purposes. Our understandings from this phase of our study provided us with a long list of requirements that a clinical data accessing system should satisfy. The intuitiveness, comprehensiveness, and flexibility of the system were identified as important points to consider when designing a computer system for accessing and interfacing clinical data.

### 4.2 Observing trauma intensive care unit work practices (qualitative studies)

The main themes of the clinical work practices that we identified from the observations include morning rounds, emergency notification, administrative work, and patient status analysis. Our findings from these themes provide us with many design insights for a clinical data interfacing system such as *MediSenseView*. The observation results and problems we have identified are summarized in Table 2 and detailed below.

**Table 2:** Summary of user groups’ work activities and the primary issues they face regarding the usage of computer systems for accessing clinical data. For each of the points, we present how these issues map to the problem statement numbers discussed in Section 5.

Group	Clinical Activity	Summary	Problem	Problem Statement
Doctors	Morning Rounds	**Identify**: Current patient status and nocturnal report overnight to review and discuss the status.	**Not available**: Identifying the physiological signal using EMR Controlling the time interval of recorded data to check detailed patient status.	1, 3
	Mortality Report Meeting	**Review**: Dead patients’ physiological signals to improve/design clinical processes.	**Not available**: Identifying the physiological signal using EMR. Using data interface in the meeting room. **Difficulties**: Limited clinical data access.	1, 2
	Emergency Notifications	**Receive**: Calls regarding drug prescription and urgent clinical decisions.	**Not available**: Identifying the patient’s status on the move. Remote access to the patient monitor.	3
Clinical Nurses	Patient Status Analysis	**Identify and record**: Patient’s status in EMR to proceed with the clinical process, to inform and call the doctor.	**Not available**: Identifying a patient’s previous status using EMR to record/archive physiological signal. **Difficulties**: Recording patient monitor data to the EMR.	1, 2
	ECG Scanning (Admin work)	**Record and print**: Abnormal physiological signal after the clinical treatment when important events occur.	**Difficulties**: Printing and pasting physiological signal using a printer located at the TICU main desk.	2
Research Nurses	Patient Status Analysis	**Collect and analyze**: Data from various previous patients to improve the quality of clinical process.	**Not available**: Identifying the physiological signal using EMR. Controlling the time interval of the recorded data to check detailed patient status.	1

#### 4.2.1 Morning rounds & mortality report meeting

**Fig 3:**
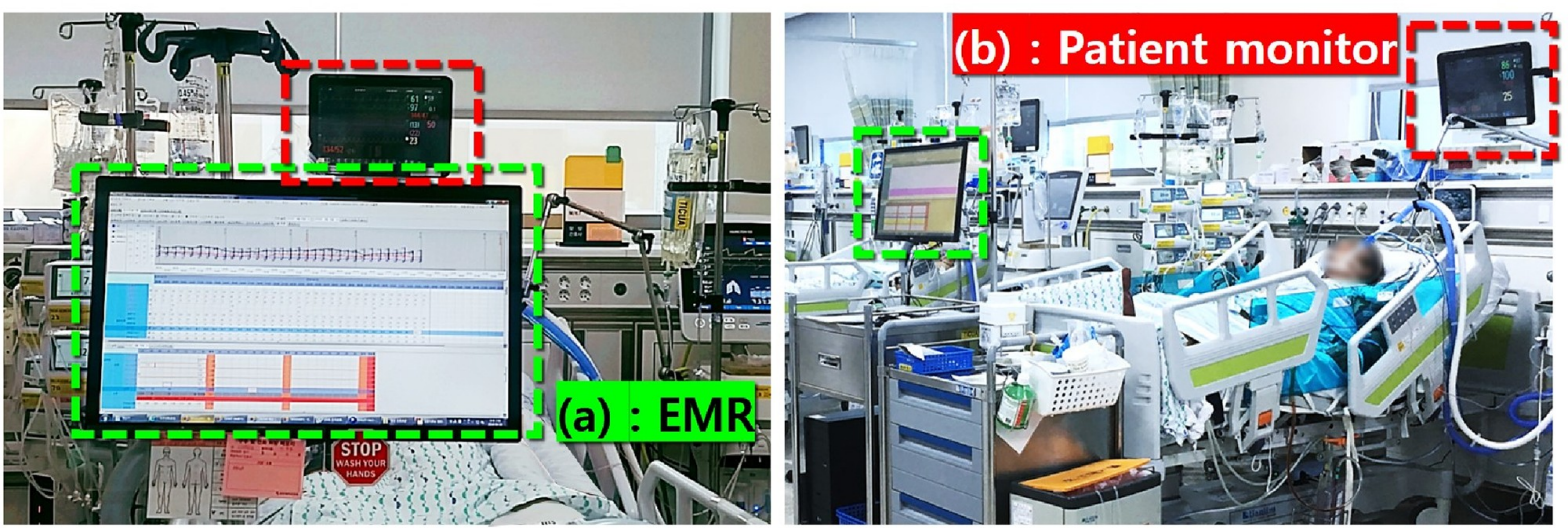
A patient bed at the target university hospital’s TICU. The computer displaying the EMR information (green box) for each patient is located in front of each TICU bed (a). The patient monitoring device (red box), collecting physiological signals (e.g., ECG, SpO2), is positioned near the head of the patient with its sensors attached to the patient on the bed (b).

During morning rounds, the EMR screen of the desktop monitor placed in front of the patient’s bed (Fig 3(a)) is reviewed. The initial analysis of the patient’s status is usually done based on the EMR’s graph plots on the patient data. However, inefficient clinical processes often occur due to the limited controllability of the EMR interface. Multiple graph plots, such as bar graphs and line graphs, overlap due to the fixed (uncontrollable) y-axis range. If two or more lines overlap, the top panel becomes very confusing to make proper observations, forcing the clinical staff to review raw numbers from the tables instead. This naturally delays the process of analyzing the status of the patient, causing inefficiency to the clinical staff.

During morning rounds and mortality report meetings, clinical staff analyze the reports from both the EMR and patient monitors. Since the EMR only shows a *summary* of the physiological signal data, to identify anomalies in the raw signals (e.g., ECG waves), the clinical staff access a separately located patient monitor (positioned near the head of the patient’s bed). Even when doing so, a staff member needs to go through the entire time series data (sequentially) to back track towards a specific time frame. Such limitations cause additional inefficiencies in the patient status analysis phase. Furthermore, for the mortality report meetings, doctors mostly depend on the paper-based reports (e.g., patient summary, physiological signals) prepared by clinical and research nurses with additional manual effort. Because the EMR and patient monitor systems are not integrated together, making comprehensive observations can be challenging. With many patients waiting to be seen, and in urgent situations where decisions need to made quickly, this delay and inefficiency and potentially erroneous manual process can be critical.

#### 4.2.2 Emergency notifications

Emergency notifications are typically issued by a nurse to a doctor when a patient is in an emergency situation or his/her condition needs to be checked promptly. Given the diverse roles of doctors (from clinical care to research), they are not always within an ICU. When receiving an emergency notification, it is important that detailed information on the patient is delivered to the doctor quickly to make proper decisions. In current protocols, clinical nurses deliver readings over a phone call as the doctors come to the ICU. During this process, human errors can occur and doctors often request for information that is not easily verbally deliverable. Furthermore, while a more experienced staff can well summarize the information to deliver, newer staff members have trouble in identifying what is more important and what is less so. In such emergency situations, a mobile interface to access the patient data can help reduce errors and make accurate decisions. Once again, the time for doctors to react to an emergency notification, from their office to the ICU, is typically less than 5 minutes; long enough for most patients to wait, but too short for many critical patients in the trauma center.

#### 4.2.3 Administrative work

**Fig 4:**
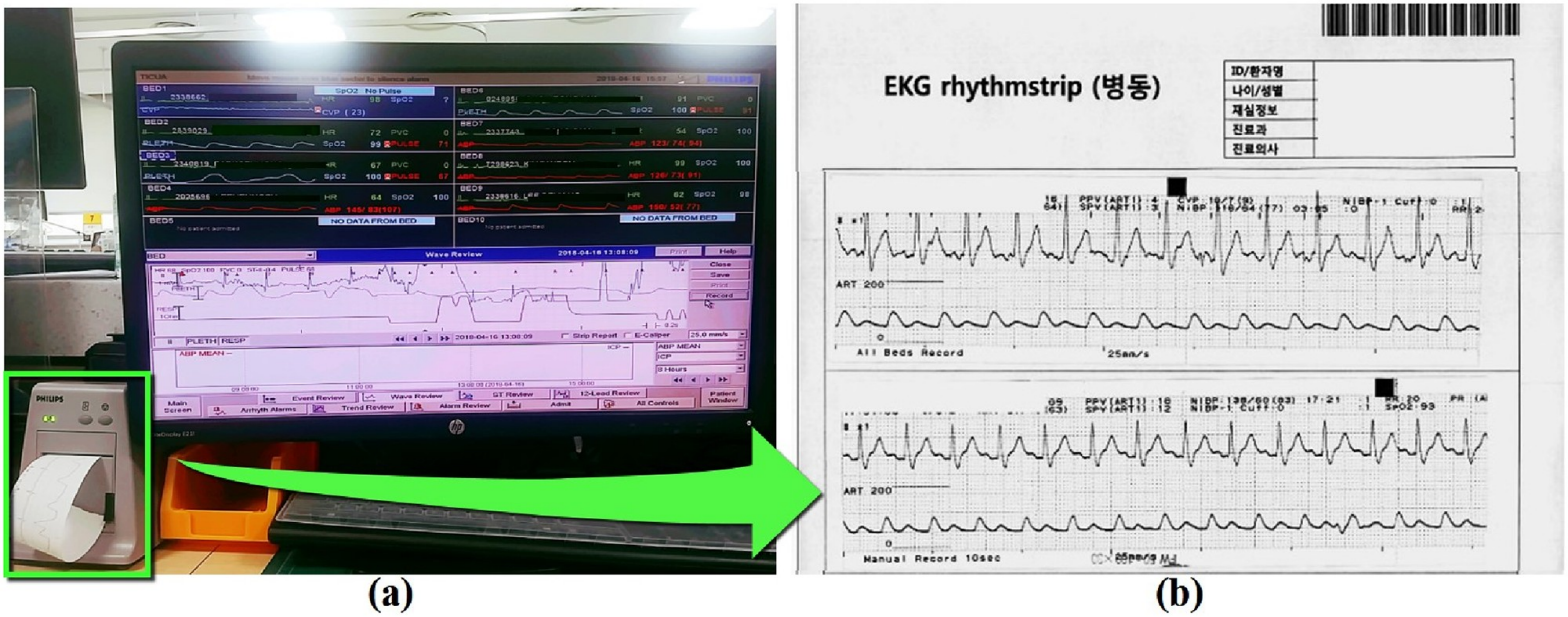
Patient monitoring interface (a) and how ECG signal data is archived on paper (b). When a notable event occurs, a clinical nurse records the time of the event and the corresponding patient condition. A PC located at the center of the TICU shows the ECG signal history for currently admitted patients. This PC is accessed to retrieve the ECG signal history. The green box in (a) shows how the ECG snippets are printed, prior to being pasted on the bar-coded paper in (b).

Clinical nurses have high workloads, which include continuous patient care as well as a great amount of administrative work. This pressure on administrative work often stresses the nurses, leaving only a small amount of time for them to focus on patient care. Under such circumstances, we want to highlight one of the most (surprisingly) manual and time consuming administrative work that we observed: ECG Record Scanning. When recording an emergency event, nurses store the patient’s physiological data by scanning the ECG wave print outs. The nurses check the event time through the EMR, access a separately located patient monitor, click on a seek button many times to access the target time frame, and print the target physiological signal plot. Then, this printed paper is glued to a barcoded paper, to be scanned and saved on the server. Fig 4(a) shows an example of how the ECG plots are printed and (b) shows how they are pasted and scanned on a paper for archiving. Such data are used in patient review meetings and mortality report meetings. ECG scanning is just one example of the administrative work that clinical nurses go through. The more manual administrative jobs the clinical staff are exposed to, the less they can care about their patients.

#### 4.2.4 Patient status analysis

To identify a patient’s status, staff members typically access the EMR dataset. However, once again, the patient’s full physiological signal history is only accessible through the patient monitors (c.f., Fig 3(b)) [13]. In most hospitals, this situation is even worse, given that the patient monitor resets (and deletes) its data regularly or when the patient is discharged. Such limitations provide reasons for ECG scanning or other manual administrative procedures, just for the sake of archiving important data. While some hospitals have recently put efforts to gather and archive such physiological data [60, 61], an interface system for effective data access for such integrated data is yet to be developed. The efforts put into setting up a physiological signal database (at a hospital scale) is expected to ease the issue of “losing” data on patient discharge, but the challenge of having two separate computer interfaces for the EMR data and the physiological signals still remains.

### 4.3 User experience with existing systems (Quantitative studies)

Prior to the interface design, we surveyed the trauma center staff on their experiences with the current interface system to identify the user’s perceived problems with the interface and what component are especially inefficient. A total of 28 clinical staff (5 doctors, 8 clinical nurses, and 15 research nurses) participated in the survey. As Table 3 shows, our survey consists of eight 7-point Likert-scale questions (1: Strongly Disagree, 7: Strongly Agree) and is divided into four main sections: **(1)** Experiences with the patient monitor, **(2)** Experiences with the EMR, **(3)** Necessity of improvement and **(4)** Necessity of data integration. We used a 7-point Likert scale for the questions to obtain relatively detailed information [62]. Based on the 7-Likert scale, we organized the questionnaire into a series of single items that were quickly readable and intuitive, taking into account the survey conducted during work hour [63–65]. Based on such questionnaire, we conducted the pre-survey to observe different needs and issues that the three stakeholders have with the current interface system. This allows us to understand their practices of using computer systems as well as design problems of such use. Participants were asked about their perceptions, opinions, and attitudes towards the systems that they are currently using and *MediSenseView* developed by us. Specifically, the principle of designing our questionnaire consisting of four main sections is as follows.

**Table 3:** Pre-survey questions used in our study. Questions were grouped into four main sections, and a 7-point Likert scale was used for the answers.

No.	Question
	**User evaluation on the patient monitor interface**
1	I think the patient monitor interface is easy to use.
2	I think the patient monitor interface helps make the clinical work more efficient.
	**User evaluation on the EMR interface**
3	I think the EMR interface is easy to use.
4	I think the EMR interface helps make the clinical work more efficient.
	**Necessity of interface system improvement**
5	I think it is necessary to improve the functions of patient monitor interface and its screen configuration.
6	I think it is necessary to improve the functions of EMR interface and its screen configuration.
	**Necessity of heterogeneous interface integration**
7	I think the integration of EMR and patient monitor interfaces is necessary.
8	I think the integration of EMR and patient monitor interfaces can help streamline clinical work.

First of all, we have distinguished between easy-to-use (e.g., low-running curve) interfaces and helpful interfaces for work efficiency in the first and second sections. Easy-to-use and efficiency are the most highlighted aspects related to clinical tasks that all clinical staff mentioned. In these sections of the questionnaire, we tried to identify the usability of the current systems because we wanted to figure out the needs of all stakeholders for the interface of *MediSenseView*. Thus, we examined, for each stakeholder, which components in the current system interface are inconvenient or need to be improved functionally. Specifically, such distinction in each section allows us to identify whether stakeholders look at the current interface in a simply difficult system to use and learn or a really inefficient system to conduct their medical protocols (e.g., caused by lack of functionality on the interface). Especially, through the first question, ‘easy to use’, in the first and second sections, we could recognize not only whether the current interface was intuitively designed, but also whether steep learning curve exists or not through the deviation between different work experiences each clinical staff has. Based on the minimized perceptual errors through these two questions in the first and second sections, we can decide which components have to be carefully and intuitively designed and which functionalities have to be added or improved. In the third and fourth sections of the questionnaire, we look specifically at whether the problem that the interface currently possesses is caused by the screen configuration and the interface’s functionality. In particular, these questions help us identify the core consideration points for the UCD-based interface system design process. Furthermore, we ask for the need for the integration of the patient monitor and the EMR interfaces. Upon completion of this pre-survey, we conducted a series of interviews based on the survey data.

**Fig 5:**
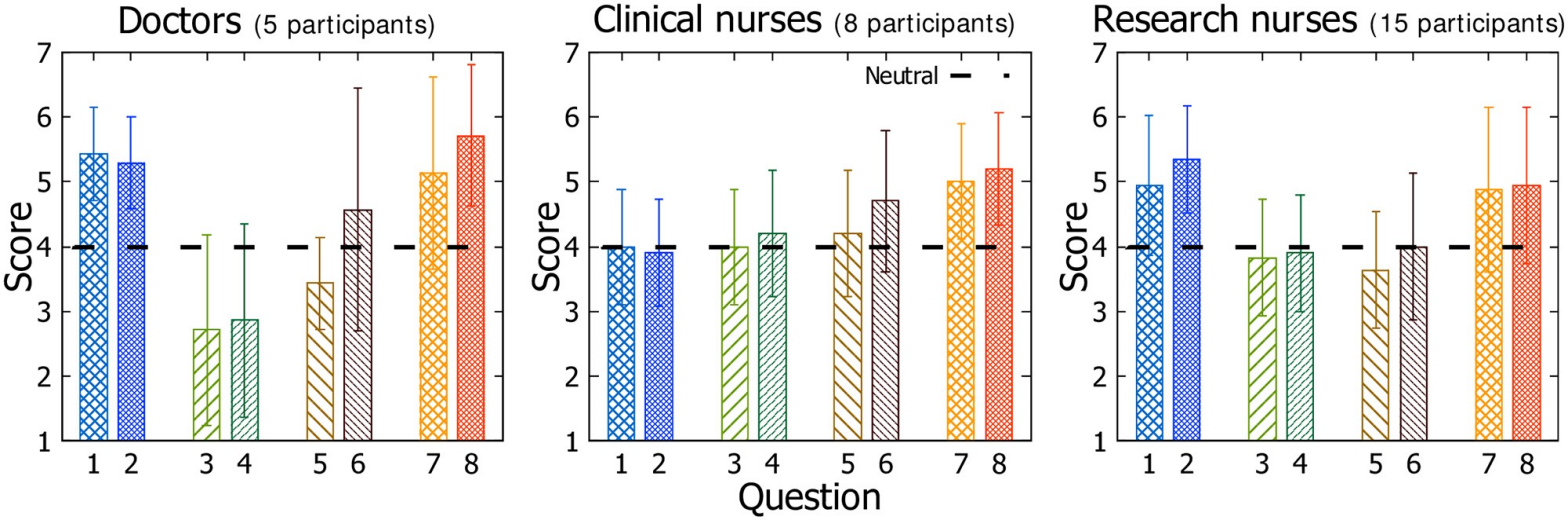
Scores (Likert-scale) for questions in the pre-study with 5 doctors, 8 clinical nurses, and 15 research nurses. Score 4 shows “neutral.”

As the survey results in Fig 5 shows, we noticed that the response scores from the clinical nurses were 4.03 and 3.92 for Questions 1 and 2 (regarding the patient monitor usage), respectively, which are lower than the doctors’ and research nurses’ responses. We note that the clinical nurses are the most active users of the patient monitors, given that these devices are only located within the TICU. It is meaningful in that they do not strongly feel that the patient monitors are easy to use. Doctors and research nurses show higher satisfaction, but we find the reliability of these results to be less than the feedback from the clinical nurses, given that the frequency of use is much higher for clinical nurses compared to the other user groups. When collecting additional comments related to these questions, clinical nurses provided feedback such as “The interface is too inefficient, and is hard to use.”, while the other user groups showed responses such as “The device control is hard to learn,” or “I usually cannot find the functions that I want to use.” Such feedback suggests that clinical nurses are aware of how to use the patient monitor functionalities, but feel uncomfortable; whereas, the research nurses and doctors are not even aware of the functions that the patient monitoring device offers.

Based on answers for Questions 3 and 4 (regarding the usability of the EMR interface), we can notice that the EMR itself is considered inefficient. Especially, the doctors’ scores are 2.71 and 2.86 for the two questions, respectively, which is lower than the other two groups. The scores provided by the clinical and research nurses are between 3.83 to 4.11. This indicates that there are conflicting satisfaction levels for the EMR between user groups. We point out that the doctors make most of their important clinical decisions based on the EMR interface and are very sensitive about the visibility of how the plots are made on the EMR screen. Nevertheless, all user groups agree on the inefficiency and difficulty of using the EMR, and suggest room for improvement.

From Questions 5 and 6, regarding the necessity of improvement to the interface systems, we observed conservative tendencies of the trauma center staff. Scores for Question 5 for doctors, clinical nurses, and research nurses were 3.43, 4.10, and 3.65, respectively. All groups had a score between 4.00 and 4.51 for Question 6, close to neutral. This interesting, given that the satisfaction levels of both the patient monitor and EMR were not high (based on answers to questions 1-4), and at the same time, their eagerness to improve and modify the interface was not noticeably high either. Subjective feedback revealed comments such as “It is true that the interfaces are inefficient. But there is nothing we can do. We just have to learn and get familiar with it. It might be good to have a better system, but we need to learn another interface, and this takes time.” Such a comment suggests that the learning curve is what blocks the staff from strongly arguing for a new interface. Therefore, we should focus on minimizing the learning curve slope when designing a new computer system for accessing clinical data.

Finally, the results for Questions 7 and 8, which deal with the need for system integration between the EMR and the patient monitor, indicate that all user groups have a strong need for integrating the EMR interface with the patient monitor data. Therefore, given their conservative stance observed earlier, we realize that we should provide an efficient and easy-to-use integrated interface that can support their clinical works and clinical processes.

## 5 Problem statements & design opportunities

Based on insights from interviews/observations and the pre-survey, we summarize three primary problems that TICU staff face when using the current clinical information systems. These problem statements were developed and refined together with clinical members participated in our study. Table 2 shows a mapping between the issues discussed above, with the problem statement numbers we define below.

### 5.1 Problem 1: Device separation (Raised by all user groups)

Using the current EMR and patient monitoring device’s interfaces, it is difficult to identify detailed/past physiological signals. In the case of the EMR, the interface only offers numeric physiological data summaries (e.g., systolic blood pressure, arterial blood pressure, respiration, heart-rate summary) on a time scale to show long-term trends. Even this discrete data is presented through non-intuitive graphs (see top panel of Fig 1), in which the plots overlap and complicate the readings. Unfortunately, the axes on the interface are not controllable and it is difficult for the staff to change the settings to better observe the graphs. As a result, for detailed observations, the clinical staff use data from the patient monitoring device directly, or access the numbers for each sensor value in the form of a table (see bottom panel of Fig 1).

Unlike the EMR, the patient monitor, located on the patient’s head (Fig 3(b)), provides real-time physiological signal data. However, as mentioned, the clinical staff tend to *not* use its full functionality. During our observation study, we noticed that the most frequent use of the patient monitor was turning on and off alarm settings and printing out ECG data. However, our brief interviews with the clinical staff, both doctors (average usage of five times a day) and nurses (average usage of more than 10 times a day), revealed that they wanted to use the device more effectively for analyzing and identifying important patterns intuitively through the interface. In addition, they noted that the physical separation of the EMR and the patient monitor devices complicated the clinical procedures since observing two data points on a same timescale is challenging. Similar issues were also raised by research nurses, who frequently analyze the trends of both EMR and patient monitor data. Having separated systems for comprehensively observed data was far from ideal for trauma center clinical protocols. Below are some quotes from our interviews with the trauma center staff.

“… we want to check patients’ disease event times and conditions during *morning rounds*. But with limited time, it is difficult to search using the current patient monitor showing a graph in 10-second increments.”

*(Doctor A)*

“While performing a variety of clinical protocols, we cannot type in all of the data in one single system.”

*(Clinical nurse C)*

“… to realize the patient status, I always check trend graphs and numeric data with medical records written by clinical nurses and doctors. But the graphs in the EMR screen are overlapped and only four of them can be selected at once. It means that the patient’s status record in EMR is in the authority of the clinical nurse. If the patient’s condition is entered every 10 minutes by the clinical nurse, I have to make predictions on the exact time of the event.”

*(Research nurse A)*

### 5.2 Problem 2: Usage inefficiency (Mostly raised by doctors and clinical nurses)

Despite having a number of computerized systems to support their work, clinical staff are suffering from a number of manual tasks. As mentioned, ECG scanning is an example, and it is unfortunate that such a task can be eased simply by having a more easy-to-use computer system interface. Specifically, this manual process is performed to satisfy two requirements. First, it is required by law that the clinical staff must make recordings of core events of a patient to confirm that the events were properly caught and treated. Second, doctors use this paper-based record in their meetings to identify any issues with the patient treatment process. Nevertheless, given that such manual processes are burdensome, reducing the frequency and overhead of manual tasks means that we can reduce the chances of human-made errors and also offer the staff with more time to look after their patients.

“During a mortality report meeting, we organize the summarized patient’s status information in the EMR and many other documents such as scanned ECG documents, surgery records, medication history, and so on. Such paper-based and summarized information reduces the work efficiency by limiting thorough analysis for a mortal patient.”

*(Doctor D)*

“… when a patient has a specific disease event, we look for the exact time it occurred using the patient monitor, and look again at the main desk ECG printer. Then we cut and paste it on paper…”

*(Clinical nurse B)*

### 5.3 Problem 3: System immobility (Raised by doctors)

The interfaces for the EMR and the patient monitors in the TICU of our interest are immobile. To be exact, the patient monitor’s location is fixed to the head side of the patient, and while the EMR interface is attached to a table with wheels, the PC uses a wall-power connection. Therefore, it is no stretch to argue that these devices possess no form of mobility. For typical clinical protocols, this is fine. However, when doctors are at distinct locations and receive emergency notifications on an urgent patient, they cannot properly access the clinical data until physically reaching the TICU. Providing interfaces on a mobile device is a tedious task for an experienced developer, but this was not yet supported by the current interface system. This problem statement was the number one issue that doctors wanted to resolve with the current hospital’s system.

“… we receive audible information on patients’ conditions verbally often but we prefer to *see* the report right away. Quite often we are in another ICU treating other patients.”

*(Doctor G)*

“… rapid clinical decision making for emergency patients would be possible if real-time patient status can be accessed remotely on the move.”

*(Doctor A)*

“During morning rounds, it is difficult to identify patients’ previous event times using the nocturnal reports. It takes too long to use the patient monitor, and the EMR graph is not sufficient to meet my needs.”

*(Doctor E)*

## 6 System design updates

### 6.1 Data used for *MediSenseView*

In order to design a system which fully integrates to the hospital environment and used by the staff, we started with a preliminary project to design a network-system to collect all physiological data from the patients and construct a bio-signal data repository [66]. A total of 100 patient monitors were connected via Ethernet connections to a server located in our hospital’s data center. All data is collected in real-time and this data is used as the base data for *MediSenseView*. In addition to the bio-signal repository, *MediSenseView* also connects the EMR server to access data and we note that our systems are integrated to present and update their data in real-time. *MediSenseView* is developed as an interactive web-based system and includes adaptive signal buffering techniques to optimize itself for the large amount of time-series data the system needs to process to provide a seamless service to the users.

### 6.2 Design update process

From the pre-surveys, interviews, and observational studies, we were able to understand the complex environment of the hospital, the clinical processes and needs of each staff group, and also how the data accessing computer systems are used at the trauma center. Based on the three primary problems and design opportunities that we identified above, we designed and developed *MediSenseView*, an interface system for accessing clinical data tailored to meet the needs of trauma center staff.

As described in Section 3, we held *three core design meetings* with *six trauma center staff members* (2 doctors, 2 clinical and 2 research nurses), as *MediSenseView* went through three main design updates.

During each core design meeting, we presented the most up-to-date version of *MediSenseView* with its new functionalities and explained how the feedback of the clinical staff was addressed from the previous meeting. We presented *MediSenseView* on a desktop, laptop and tablet device, and asked the staff to freely use it in their tasks. We answered their questions and collected additional feedback (by taking notes and voice recordings), which was used for the next system design update iteration. Additionally, in order to gather short-term feedback between the core meetings, we frequently held intermediate meetings with a subset of the members. Specifically, at design meeting *N*, we presented the resulting working-prototype from meeting *N* − 1, and we did a paper sketch-based mockup at the end of each meeting based on the received feedback to confirm new design directions before its actual implementation. The detailed discussions and feedback from each design meeting (first to third) can be found in Appendix 1.

To effectively apply the collected feedback from our core and intermediate meetings in the system redesign process, *MediSenseView* was developed and continuously installed in the hospital and was designed as a web-based system that interconnects with the hospital’s data server. Having the device at the hospital allowed the clinical staff to offer continuous feedback on the system. Being a web-based system implemented in Java and JavaScript, allowed the system to be applied in various user platforms.

**Fig 6:**
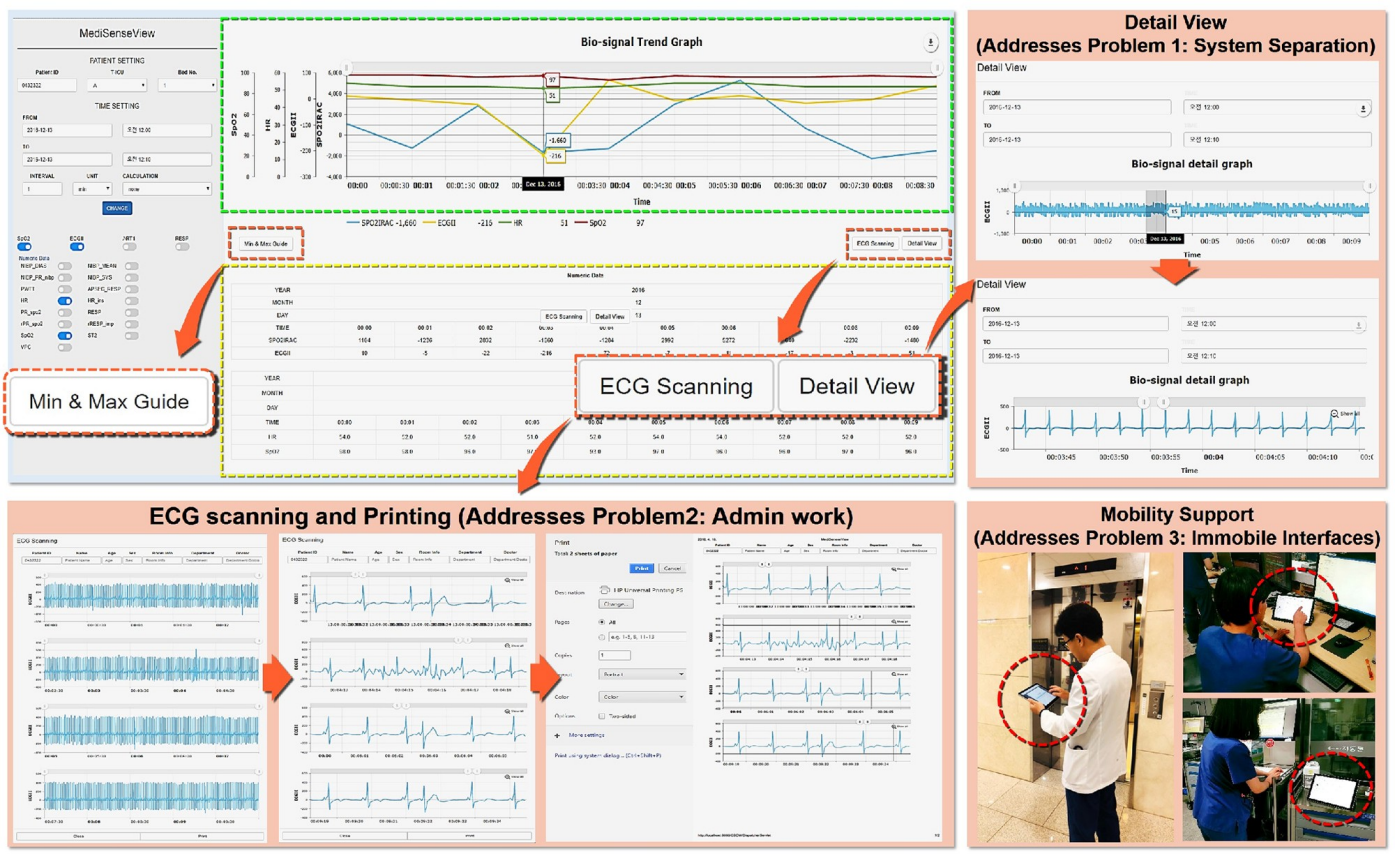
Final version of the *MediSenseView* prototype. This version was used for the pilot deployments and post-deployment user study. The design process was a result of thee core design meetings with the trauma center staff. To address the core issues for the existing interface systems, *MediSenseView* combines the data exposed from two separate interfaces, supports mobile usage and eases manual routine tasks. We present how the system reacts to different button-presses and how the final version of *MediSenseView* resolves all of the three core problems we have identified.

As a result of the series of meeting and re-designing process, as Fig 6 shows, *MediSenseView* is designed with top and bottom panels, consisting of a controllable graph plot and a table with numerical values, respectively. Note that to observe detailed physiological signals, a button still needs to be clicked (e.g., Detail View button). Nevertheless, the EMR data and patient monitor data are combined in a single interface (resolved Problem 1: Device Separation). Automated ECG scanning features were added to ease the burdens of clinical nurses (resolved Problem 2: Usage Inefficiency), and we optimized the system to work well with mobile platforms to provide doctors with mobility support (resolved Problem 3: System Immobility). Moreover, in the trend graph plots, we designed the bullet and vertical lines (hover effects) so that the staff can observe the exact numerical value of the physiological signals by placing the mouse at the desired timestamp. Another feature that the staff were pleased with was the Min & Max Guide feature. This was implemented with reference to some feedback from the design meetings, allowing the clinical staff to configure an audible and visible alarm when physiological signals go above or lower than a preset threshold, a feature that needed to be done by accessing the patient monitoring device each time.

Overall, we would like to emphasize that the design of *MediSenseView* was a result of a series of comments and feedback received directly from the actual users of the system. This continuous feedback processes revealed a series of information that could not be caught in formalized interviews, surveys or indirect observational studies alone.

## 7 System evaluation

Using the final version discussed above, we deployed *MediSenseView* in the trauma center as part of a 1.5 month pilot deployment to measure the usability through pre- and post-survey comparisons and performed a post-study to observe the acceptability of the new clinical data interface system to the staff members at our university hospital’s trauma center. Note that we designed *MediSenseView* as a workable real-time interface connected to the hospital’s physiological signal data server, covering seven functioning ICUs consisting of three trauma ICUs, one medical ICU, one emergency ICU, and two surgical ICUs [67]. Since 2017, we have collected physiological signal data from a total of 30,593 patients, which add up to 14.1 TB of total data. Using this back-end server that gathers real-time physiological signal data, we deployed MediSenseView in accordance with the approved IRB period (AJIRB-SBR-OBS-16-507, Mar 8 2017—Dec 31 2019) to conduct surveys and interviews. During the 1.5 months of field testing, *MediSenseView* was used as a supplementary interface to the clinical staff for serving ∼400 patients admitted to the trauma center ICU. All physiological signals used in the interface were collected through the real-time data collection interface via the hospital’s physiological signal data server. All interviews and surveys were approved by Ajou University Hospital Institutional review board (AJIRB) and written consent forms were exempt through the IRB (only requiring verbal consent).

### 7.1 Post study details

We had two major objectives in conducting the evaluations. First, we gave a specific task to the clinical staff to see how they responded to the system with a minimal amount of learning. We developed and refined the task with the clinical staff (who did not take part in the evaluation) and confirmed that we offer a usable task that would actually be performed in real operation. Specifically, we provided a set of patient data and asked the staff to identify patients with abnormal heartbeats. From this, we wanted to verify the efficiency and effectiveness of *MediSenseView* with the real users and make sure that the learning curve was not too steep. Second, we asked the staff to answer a post-survey. The post-survey consisted of eight questions as presented in Table 4. Questions 1-2 asked about the staff’s experience with *MediSenseView* compared to the patient monitoring device’s interface. Questions 3-4 asked about the experience in *MediSenseView* compared to the current EMR interface and we ask questions on the work efficiency for doctors (Question 5) and nurses (Question 6), respectively. Questions 7 and 8 focus on capturing users’ experience in the task that we provided. A total of 28 clinical staff members (i.e., 5 doctors, 8 clinical nurses, and 15 research nurses) participated in the post study. The same set of members also participated in the pre-survey; thus, they could provide direct feedback on and their explicit experience in using *MediSenseView*.

**Table 4:** Questions used in the post-survey. Answers were provided in 7-point Likert scale.

No.	Question
	**Comparison between** *MediSenseView* **and the patient monitor interface**
1	*MediSenseView* is easier to use than the patient monitor interface.
2	With *MediSenseView*, I can work more efficiently than using the patient monitor interface directly.
	**Comparison between** *MediSenseView* **and the hospital EMR interface**
3	*MediSenseView* is easier to use than the EMR interface.
4	With *MediSenseView*, I can work more efficiently than using the EMR interface directly.
	**Experienced efficiency for each user group**
5	[Clinical and Research Nurses only] With *MediSenseView*, I can do my work more efficiently.
6	[Doctors only] With *MediSenseView*, I can effectively identify a patient’s status on the move.
	**Experience on task completion**
7	When looking for abnormal signals, I can easily find the occurred time of the event.
8	If I know the timestamp of an abnormal signal, I can easily find abnormal signals.

### 7.2 Post study results

**Fig 7:**
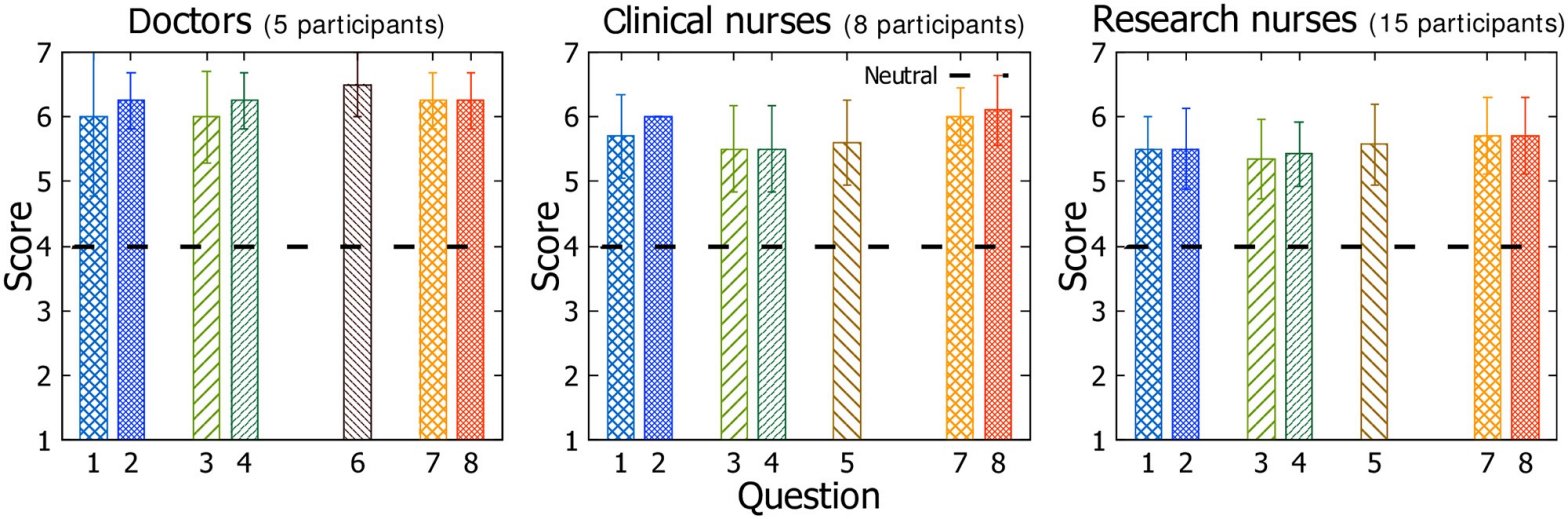
Scores for the multiple choice questions in the post survey with 5 doctors, 8 clinical nurses and 15 research nurses. Score 4 represents “neutral” in a 7 point Likert-scale.

The clinical staff’s overall perception to *MediSenseView* was positive. As illustrated in Fig 7, most responses were either close to 6.0 or above on a 7-point Likert scale.

Specifically, in Questions 1 and 2 (patient monitor data-related), while the doctors and research nurses were already quite satisfied with the existing patient monitor interface (based on the pre-study results), the result of the post-survey shows that they are relatively more satisfied with *MediSenseView*. Such positive result is even more noticeable in the case of the clinical nurses. Being a group that frequently uses patient monitors compared to the other groups, they show the most positive change in using *MediSenseView* versus the patient monitor.

Based on the results for Questions 3 and 4 (EMR data-related), the results from the doctors show 6 and 6.25, respectively, in the post-study. Considering that they frequently make clinical decisions based on the EMR data and are very sensitive about the visibility of the plots on the EMR screen, the scores show that the user-centered design of *MediSenseView* supports their requests and is easy to use. This increasing trend in satisfaction in using *MediSenseView* also holds for the clinical and research nurse groups as well. These overall positive results regarding the use of *MediSenseView* also suggest that the learning of the interface usage was not difficult for the users. This again, we believe is an effect of continuously capturing the feedback through our three core design meetings.

Finally, answers to question 6 from the doctors suggest that they find *MediSenseView* effective enough to access patient data on mobile platforms (Question 6) and feel confident in identifying abnormal signals (Questions 7 and 8) using *MediSenseView*. The clinical and research nurses showed similar responses. While their responses are not as noticeably high as the doctors’ (Question 5), when comparing with the results obtained in the pre-survey, they were positive about *MediSenseView* with respect to the ease and increased efficiency of their work. Both the clinical and research nurse groups similarly showed a great confidence in completing the given tasks (clinical nurses: 5.90(Mean)±0.54(SD); research nurses: 5.59±0.59).

## 8 Discussions and future directions

Based on our experiences in performing interviews, surveys, and observational studies with the clinical staff, and also from our experiences in designing and implementing *MediSenseView*, we outline a few interesting points for further discussion.

### 8.1 Design suggestions

Our post-study using *MediSenseView* resulted in another set of interesting feedback that we can consider as part of future work. Below are some points worth sharing.

- Research nurses asked if there could be additional support for easily integrating algorithms for clinical data analysis. Given that this user group focuses on extracting clinical insights from the raw data, this was a customized request for their specific purposes.
- A subset of the doctors that participated in our post-survey was satisfied with *MediSenseView* and hoped the system could be portable to a smartphone rather than a tablet. This would require major changes to the current layout, but given that this user group prefers to utilize *MediSenseView* “on the move,” we see this as an important feedback and plan to address this in our next revision.
- Doctors and clinical nurses asked that we limit the zoom-in capabilities of *MediSenseView* for physiological signals to a contextually-effective range. The current implementation of *MediSenseView* allows the physiological signals to be zoomed up to a single-sample scale. However, the users noticed that when observing physiological data, short-term trends can be important, but single-sample scale visualization can only complicate the users.

### 8.2 Inter-hospital and inter-department extensions

Our prototype interface, which is systematically designed through a series of interviews and surveys from a trauma care unit, is designed to provide the staff with a more intuitive environment in analyzing patients’ information. While we consider the study to be comprehensive, the question of design generality yet remains. “Will this system design be optimal for other trauma centers?” “Can we apply the same system to other clinical departments?” These questions still remain unanswered.

On an inter-hospital perspective, our observational studies and discussions suggest that the work process will not differ dramatically in other trauma centers. Many of the tasks are already part of a gold-standard clinical protocol, and only a few differences may arise given that other hospitals have slightly-different interface systems from other vendors. Nevertheless, our informal verbal discussions with three other trauma center personnel in the region suggests that similar issues, such as data integration [27, 68, 69], mobile device support [24, 70], and manual administrative workload [71, 72], still hold. While preferences on interface design/layout and the input data formats may differ, the fundamental visualization issues that *MediSenseView* tries to address can be considered valid. Nevertheless, a validation phase on how the clinical protocols are applied and how interfaces interact with the system users in different hospital settings still needs to be validated prior to deployments.

The harder question to answer is the issue of inter-department applicability. We can intuitively think that a requirement of an orthodontist can be very different from trauma care personnel. On a practical hospital administration perspective, not only is it cost-effective to service a single interface system across all departments, but data sharing can also be an issue. First, given that the priorities of patient status information that doctors from different departments strive to first identify are different [52], having a single interface may not be ideal. In fact, this is the main reason we decided to design *MediSenseView* so that the interface system is optimized for TICU environments. Therefore, deploying the system across different departments is part of our future work and we are currently in the process of gathering requirement lists from related, but different clinical departments (e.g., emergency medicine). Regarding the second issue of data sharing, while not a feature offered by *MediSenseView*, the hospital we perform our experience in already offers a unified data format used across departments, which eases the development of interfaces easier, given that the backend implementations will remain the same.

### 8.3 Paper-based data vs. computerized data

The current clinical protocols are typically performed using a combination of computer-based patient data and handwritten documents. Doctors will often carry printed charts of the patient, which they use to “scribble” their thoughts on, and this plays an important role in the final decision making process [73, 74]. Furthermore, as we saw in the case of ECG data scanning, paper documents are still frequently used in meetings when the clinical staff discuss ideas on how to treat a patient. The procedures that *MediSenseView* tries to improve, mostly focus on the computerized data within the clinical environment. Integrating paper-written knowledge (data) as part of a computerized system remains as a task to resolve [75, 76].

### 8.4 Better interfaces? or better analysis algorithms?

A handful of previous work focus on using the data used in hospitals to make predictions and assistive systems for patient diagnosis [5, 6]. We agree this is a valid direction of research. On the other hand, our approach is to provide the clinical and research staff with a better data visualization so that we can maximize the human-side of clinical intuition and data analysis. We believe that using the data available in hospitals today, both directions of research are meaningful. While *MediSenseView* currently (mostly) focuses on providing clinical staff with a new way of observing and utilizing the data, we hope that their long-term usage patterns with our system and the new intuitions on the data can offer novel clinical insights in designing better (automated) clinical data analysis algorithms as well.

### 8.5 Physiological signal data availability

We were fortunate enough to interface *MediSenseView* with two important data sets: the EMR and the physiological signal database. This was essential to resolve the first problem of system separation. While EMR data is available in many hospitals, only a few hospitals keep a real-time database of the physiological signal data. Typically, physiological signals are observed through the patient monitor device and are discarded once the memory available on these devices are full, or when the patient is discharged. This is usually due to the massive amount of data that these devices produce and the extra efforts to connect patient monitors with the hospital’s data vaults [77, 78]. The hospital that we work with has gone through recent renovations so that all physiological signal data are saved for future clinical research (IRB-approved). Nevertheless, such a system is a collaborative effort with clinical staff and engineers that need to be managed on a hospital-scale. Our studies with the hospital staff suggest that integrating physiological signal data with EMR can be very beneficial. We argue that such findings should be carefully considered in many hospitals to offer a data-integrated environment.

### 8.6 Practical usage efficiency

#### 8.6.1 Beyond system development

Most prior studies on the interface and system design in the hospital context have primarily focused on user and environment understandings, and/or system development. However, an in-depth investigation on user experiences when utilizing the system in actual use cases have not yet been studied in most prior work. Although a survey using the designed Activity-Based Computing (ABC) framework was conducted in Badram’s paper [42], this framework considers a new interface design based on activity theory and was evaluated through an only qualitative measure with five pre-defined scenarios. In addition, in the same work by Badram, 11 workshops were conducted for evaluation and design, but the paper did not give detailed information about what kind of opinions were presented in each workshop, whether the opinions were reflected in the interface design process, etc. Four of them were scenario-based evaluation workshops, and as outcomes of the workshops, there was no discussion about user experience or additional important design recommendations. On the other hand, our work is different in that we not only designed and developed *MediSenseView* through a series of interactions with clinical stakeholders that reflect their current work practice and needs, but also present empirical evaluation and user experience through each stakeholder’s practical use of *MediSenseView*. The results of the deployment study indicate that clinical doctors evaluated great convenience and usability regarding the mobility of *MediSenseView*. Interestingly, this result is somewhat contradictory to the one observed in Badrum’s activity roaming study [42], where the use of PDAs and tablets was negatively evaluated by clinicians. Clinical and research nurses showed positive feedback especially on system integration and work efficiency. In addition, in practice, these staff members mentioned for the need for customized functions to quickly observe real-time patient conditions (e.g., simple statistics). This was an additional requirement identified through our real-use deployments in the TICU and we plan to add such factors into the system as part of future work when the system is authorized for long-term use in the hospital.

#### 8.6.2 New system development vs. expansion of existing systems

Most studies of system development (either with or without UCD) in the clinical context have been done in the direction of identifying user requirements and creating a “new” system [41, 49]. In contrast, MediSenseView not only reflects the requirements related to users’ inconvenience to the existing system but also extends the existing system. We noticed that most clinical staff in our study are sensitive to learning a new system, showing conservative responses. Based on this, we focused on identifying user needs/requirements and developing a system that integrates the use and practice of the existing systems (i.e., patient monitor, EMR). Perhaps because of this, most participants quickly adapted to the use of MediSenseView and showed positive evaluations of the system. Note that we are not emphasizing that our approach is better than the ones used in other prior studies. Instead, we want to highlight that it is important to provide a system that minimizes the learning curve for the busy medical staff [52], as discussed in the development of a new system interface through participatory design, which requires the consideration of various needs of different users or groups in the process of system design. One interesting finding in this study is about a trade-off for reconciling different information needs, which remains as a discussion point to solve and discussed in other studies as well [55]. In this sense, our approach of adopting and expanding existing system functionalities to the design of MediSenseView may be more effective in terms of obtaining consensus from different stakeholders. Note that in some settings, new clinical devices can be introduced to the clinical environment. If the new device introduces the same modality (e.g., ECG device change among different vendors) given that the hospital database exploits a unified format for all ECG vendors, the integration to *MediSenseView* will not be a major issue as it exploits data streams provided from the server. For modalities currently not supported, *MediSenseView* may show limitations. First, if the data can be presented as time-series numerical data, we can easily present the data using the plot formats that we currently present on *MediSenseView*’s screen. On the other hand, if this new modality is in an entirely different and currently unsupported format (e.g., audio), this would require changes to the interface design as exploiting such data will most likely lead to change in clinical protocols. Nevertheless, while not ideal, we can achieve this by adding a functionality to open a new page that displays the new modality. Designing this additional page of data would be a sub-optimal way of supporting new modalities. While being a current limitation of *MediSenseView*, this is a fundamental challenge for most non-interactive interfaces.

## 9 Conclusion

Trauma care units at any hospital is unarguably one of the busiest departments, repeatedly taking care of patients on the line of life and death. Given its special circumstances, the design requirements for clinical data interfacing systems of staff members working in such urgent environments can be different from that of other departments. The goal of our year-long study was to provide these staff members: doctors, clinical nurses and research nurses in the trauma center, with a clinical data interface system customized for their requirements, so that better clinical decisions could be made efficiently. Our study identified that, indeed, today’s interface systems for clinical data are not up-to-par with the specific requirements and protocols of the trauma center. The separation of data over multiple heterogeneous platforms and interfaces, the lack of mobility support, and the inefficiencies that arise from manual administrative work suggest for a new user-centered interface system. *MediSenseView* is our answer to address such problems. The design of *MediSenseView*, a user-centered interface system, is based on a series of meetings with the potential users of the system. We observe its user acceptance levels using a post-survey to report that *MediSenseView* was well received by the clinical staff to improve the efficiency of clinical protocols in the trauma care unit. Based on our long-term experiences, we outline a number of remaining research directions for providing a better work environment for our partners in the trauma center. We envision this study as a stepping stone towards contributing additional customized, user-centered computing technologies for urgent clinical environments.

## Appendices

### Appendix 1: Details on design meetings

Using Appendix 1, we present details on the three-stage design meetings we held with the clinical staff members. As discussed in Section 8, we conducted a total of three design meetings and the following discusses detailed findings and changes made to the interface design.

#### Initial design meeting

**Fig 8:**
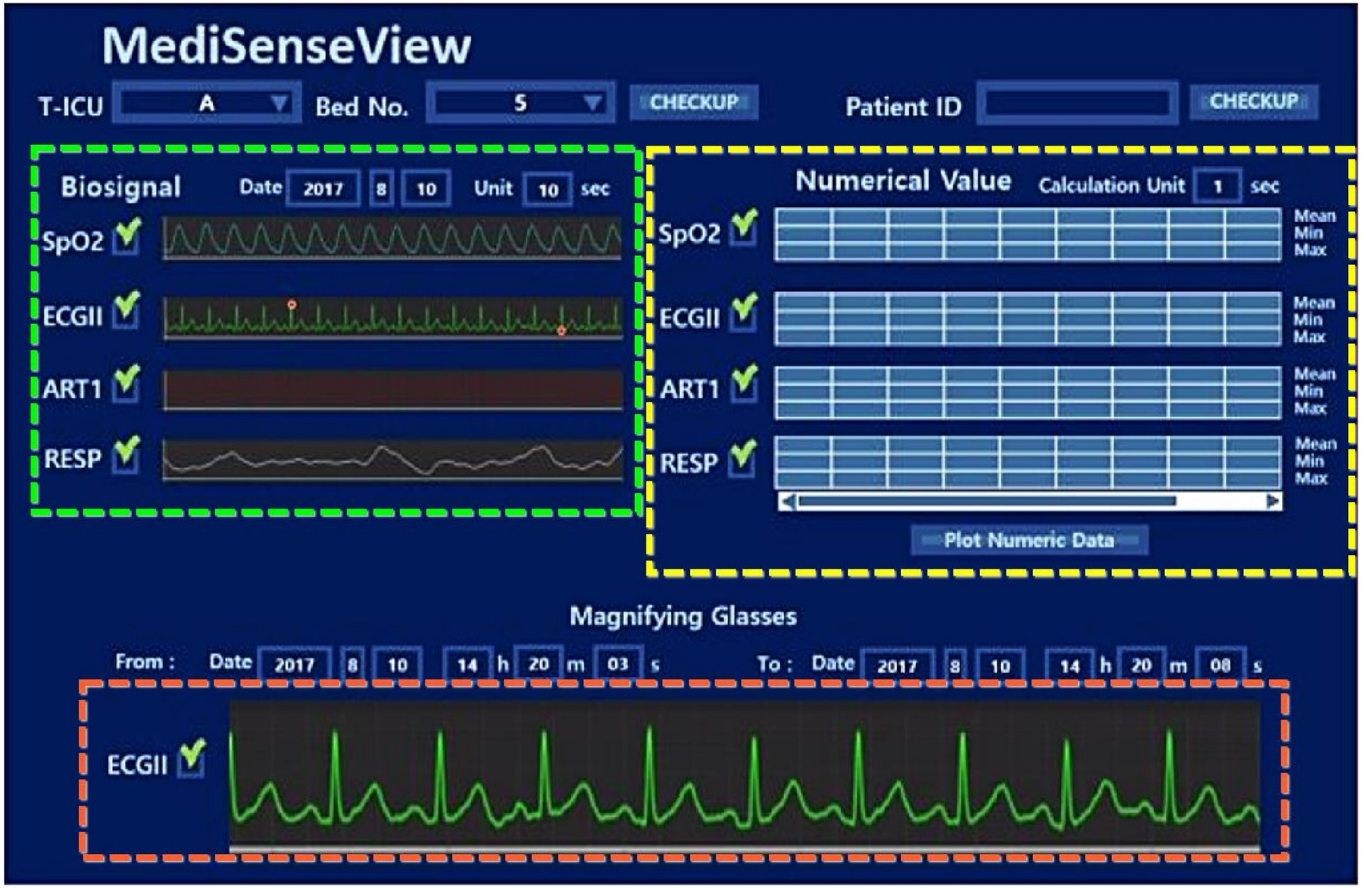
The first prototype of *MediSenseView*. We used this version for discussions in the first design meeting.

Our first version of *MediSenseView*, Fig 8, provided *a* solution to the three problems we have identified. Specifically, in this initial prototype of *MediSenseView*, we focused on the integration of the patient monitor data and the EMR. The three core components implemented in this initial design include: (1) trend plots for the values in the EMR data and physiological signal data (green box in Fig 8), (2) the numerical values from the EMR database (yellow box in Fig 8), and (3) a detailed view of a selected patient’s physiological data (orange box in Fig 8). Using this initial implementation, we received a long-list of feedback from the clinical staff. The following are some of the insightful comments we received:

“… when using the mobile device, showing the trend plot as the main content of the first (initial) screen will be better in helping me read data from the plot.”

*(Doctor A)*

“… it would be nice to design the plots to be more intuitive. Numerical values and the detailed physiological signal data view are of less importance to us on the first screen,”

*(Research nurse A)*

“… we want to see the EMR data first. would be better to use the button as an additional function than to show detailed physiological signals.”

*(Clinical nurse A)*

#### Second design deeting

**Fig 9:**
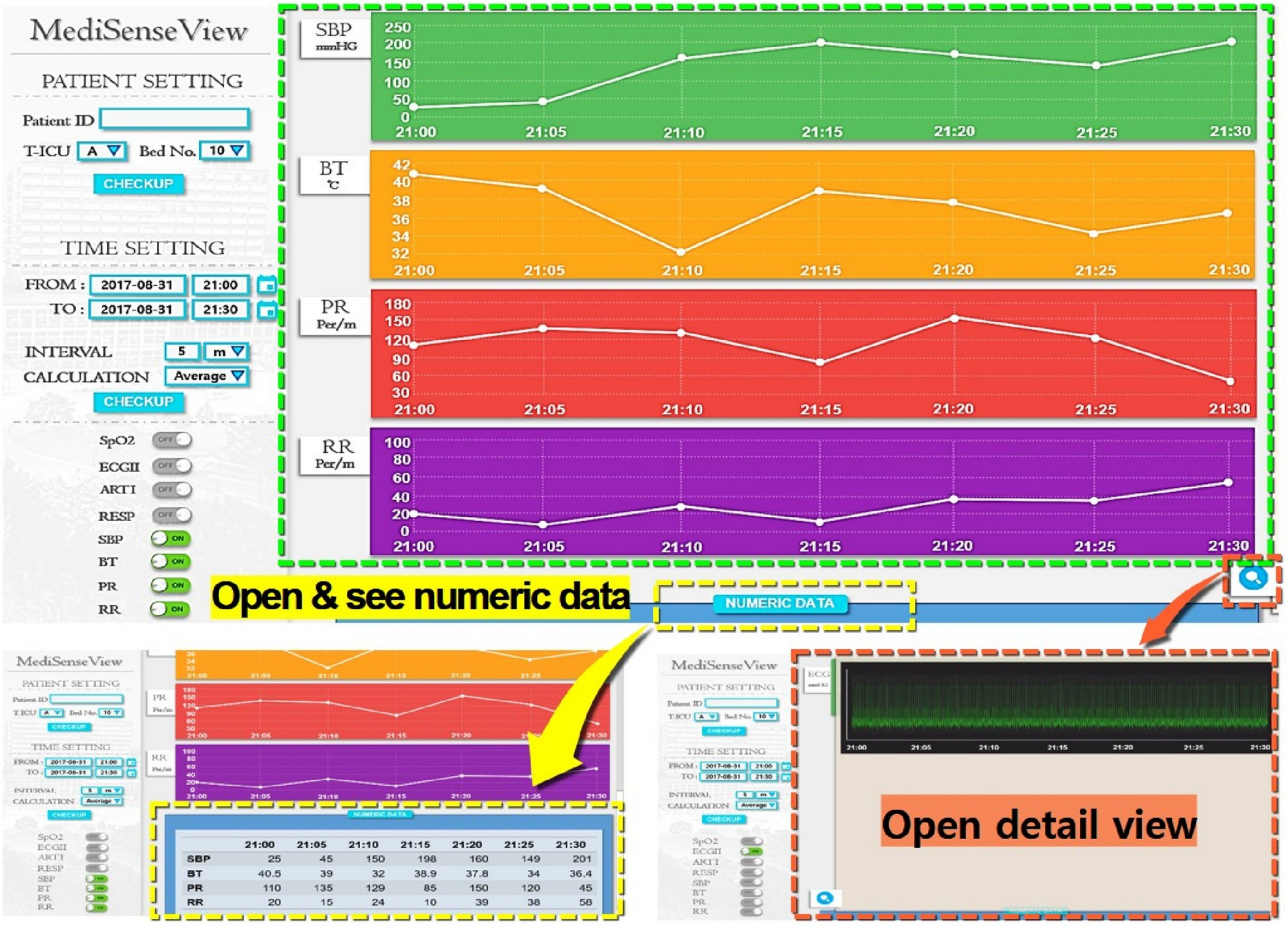
The second version of *MediSenseView*. This version is designed based on the feedback from the first design meeting with the trauma center panel. We present how the screen expands as different buttons are pressed.

Using the initial set of feedback, the second version of *MediSenseView* aimed to provide an interface that focuses the first screen to the trend plots for the physiological signals and EMR data (green box in Fig 9). We also allowed the users to select the desired modalities for graph plot display. The numerical values and the detailed view of physiological signals were minimized so that they only open when buttons were clicked (yellow and orange boxes in Fig 9). Our next meeting with the hospital staff, using this revised system, pointed out that the plots needed to be better correlated in time. Given that our first and second revisions had separate plots for each data modality, correlating them over the x-axis (i.e., time) seemed to be a concern for the users. In some sense, the original EMR plots on the top of Fig 1 show a single graph plot with all the values’ trends. While staff members thought this as an ineffective way of representing data, the x-axis correlation was still considered important. Some of the comments were as follows:

“I’d like to compare different types of physiological signals, like ECG and blood pressure, at the same time,”

*(Doctor B, Research nurse B)*

“Considering the variety of physiological signals, it would be nice if the buttons on the left bottom were horizontal than vertical,”

*(Clinical nurse B)*

“I want to have a list of the most frequently used buttons on the left bottom, like a history function.”

*(Clinical nurse A)*

#### Third design deeting

**Fig 10:**
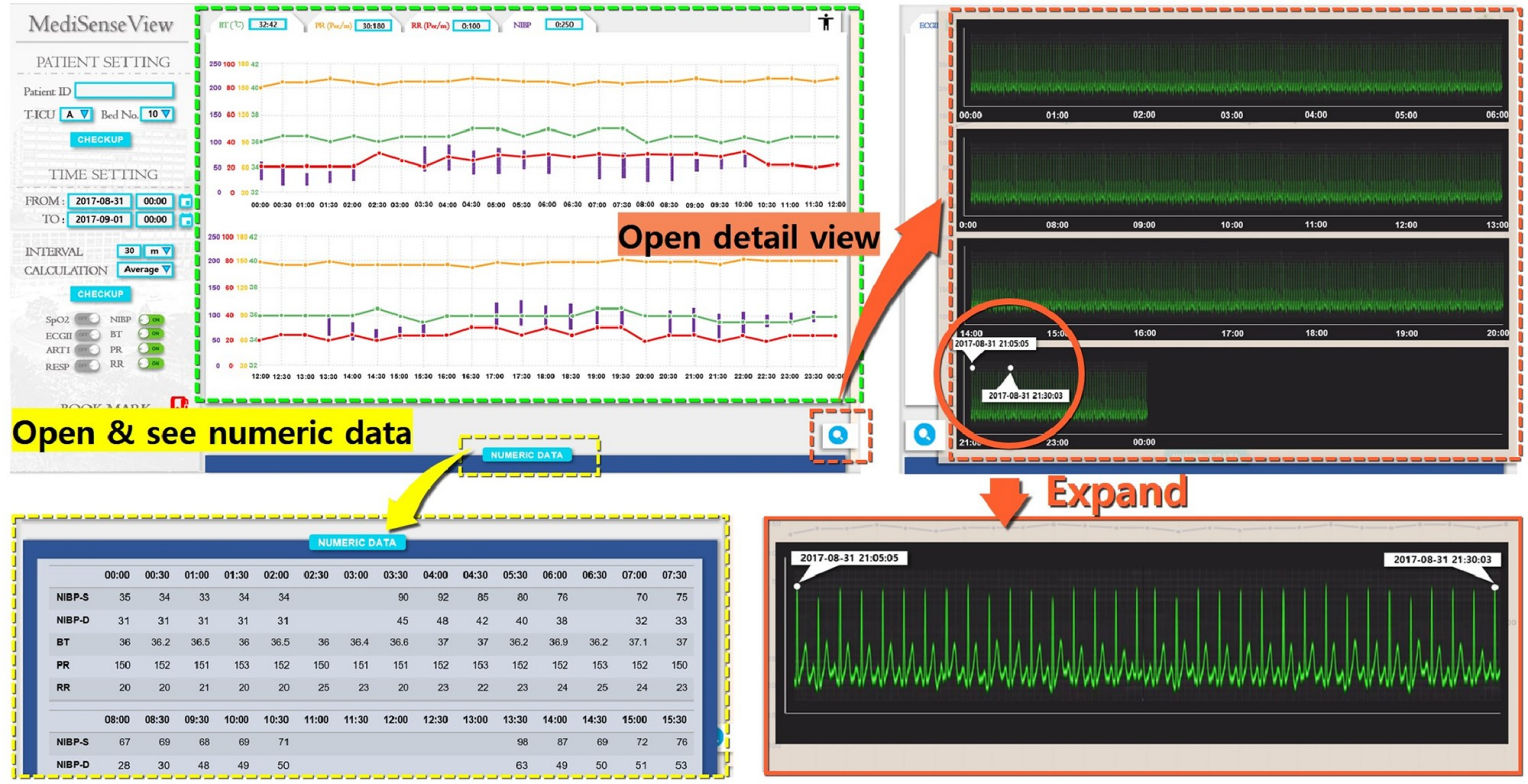
The third revision of *MediSenseView*. This version is designed based on the feedback from the second design meeting with the trauma center panel. We present how the screen expands as different buttons are pressed.

The third revision of *MediSenseView* took the aforementioned comments in consideration, and combined the plots to present the information in a single graph. Nevertheless, to resolve the inefficiency of the original EMR interface, we made sure that the y-axis was adjustable so that plots do not overlap and complicate the users (green box in Fig 10). As the yellow and orange boxes show in Fig 10, the numerical data and detailed views were kept minimal on the display. Using this design, we received another set of user feedback during the third and final core design meeting. Here, surprisingly, the staff members asked that the numerical values come back to the first (initial) screen. This was an interesting feedback given that such a design would make *MediSenseView* look similar to the original EMR interface (Fig 1). Nevertheless, they indicated that the added features of adjusting the plot ranges, controlling the plot modalities, and the integration with the patient monitor would be extremely helpful in improving their clinical practice. Some of the feedback from this phase of interviews are as follows:

“…, I still want to identify different trend graphs in one box,”

*(Research nurse B, Doctor A)*

“… having a history function that shows a list of frequently checked physiological data would be great.”

*(Clinical nurse A, B)*

### Appendix 2: Abbreviations used in this work

Below is Table 5 that summarizes the abbreviations used in this work.

**Table 5:** List of abbreviations used in this work.

Abbreviation	Description	Location
**TICU**	Trauma intensive care unit	• Figs 2–4
		• Table 2
		• Section 1, 2.3, 3, 3.1, 3.4, 4.1.2, 4.1.3, 4.3, 5, 5.3, 8.2, and 8.6.1
**EMR**	Electronic medical record	• Figs 1 and 3
		• Tables 2–4
		• Section 1, 2, 3.1, 3.3, 4, 5, 6, 7.1, 7.2, 8.5, and 8.6.2
**ICU**	Intensive care unit	• Table 1
		• Section 1, 2.1, 2.2, 3, 4.1.1, 4.1.2, 4.2.2, 5.3, and 7
**UCD**	User-centered design	• Section 2.2, 2.3, 3.3, 4.3, and 8.6.2
**HCI**	Human-computer interaction	• Section 2.1, 2.3
**CSCW**	Computer supported cooperative work	• Section 2.1
**GUI**	Graphical user interface	• Section 2.1
**IRB**	Institutional review board	• Table 1
		• Section 3, 4.1.3, 7, and 8.5
**ECG**	Electrocardiogram	• Figs 3 and 4
		• Table 2
		• Section 3.1, 4.1.2, 4.2.1, 4.2.3, 4.2.4, 5.1, 5.2, 6.2, 8.3, and 8.6.2
**CT**	Computer tomography	• Table 1
		• Section 4.1.2
**MRI**	Magnetic resonance imaging	• Table 1
		• Section 4.1.2
**SD**	Standard deviation	• Section 7.2
**EHR**	Electronic health record	• Section 2.1, and 2.3
